# Targeting Cadherin-17 Inactivates Ras/Raf/MEK/ERK Signaling and Inhibits Cell Proliferation in Gastric Cancer

**DOI:** 10.1371/journal.pone.0085296

**Published:** 2014-01-20

**Authors:** Zhaohu Lin, Chao Zhang, Meifang Zhang, Danqing Xu, Yanfen Fang, Zheng Zhou, Xiaolong Chen, Ning Qin, Xiongwen Zhang

**Affiliations:** 1 Department of Discovery Technology, Roche Pharma Research and Early Development China, Shanghai, China; 2 Shanghai Engineering Research Center of Molecular Therapeutics and New Drug Development, East China Normal University, Shanghai, China; Institut de Génétique et Développement de Rennes, France

## Abstract

Cadherin-17 (CDH17), one member of 7D-cadherin superfamily, was overexpressed in gastric cancer (GC) and was associated with poor survival, tumor recurrence, metastasis, and advanced tumor stage. So far the cellular function and signaling mechanism of CDH17 in GC remains unclear. In this study, we showed that over 66% of GC cell lines (20/30) were CDH17 positive. Tissue microarray (TMA) assay showed that 73.6% Chinese GC tissues (159/216) were CDH17 positive, while 37% respective adjacent normal tissues were CDH17 positive. Knockdown of CDH17 inhibited cell proliferation, migration, adhesion and colony formation, and also induced a cell cycle arrest and apoptosis in AGS human GC cells. On the other side, overexpression of CDH17 facilitated MGC-803 GC tumor growth in nude mice. Antibody array and Western blotting assay demonstrated that knockdown of CDH17 in AGS cells down-regulated integrin β series proteins, further inactivated the Ras/Raf/MEK/ERK pathway and led to p53 and p21 accumulation, which resulted in proliferation inhibition, cell-cycle arrest and apoptosis induction. Collectively, our data firstly demonstrate the capacity of CDH17 to regulate the activity of Ras/Raf/MEK/ERK pathway for cell proliferation in GC, and suggest that CDH17 can serve as an attractive therapeutic target for future research.

## Introduction

Gastric cancer (GC) is ranked as the second leading cause of global cancer mortality and the fourth most common cancer worldwide [1], [2]. The median survival time of GC patients is 7∼10 months. Most patients with GC present with late-stage disease with an overall 5-year survival of approximately 20% and objective response rates to conventional chemotherapeutic regimens range can be improved from 20% to 40% [1], [3]. Currently, cisplatin-based therapy is still widely used in clinical settings for advanced and metastatic GC. In addition, for HER2-neu overexpressing gastric adenocarcinomas, trastuzumab (Herceptin) in combination with chemotherapy prolongs the median overall survival from 11.1 months (chemotherapy alone) to 13.8 months [4]. Considering the high mortality rate of GC, there is still huge unmet medical need to find the sensitive and reliable biomarker for early diagnosis of GC and potent therapeutic target for treatment of GC.

CDH17, one member of 7D-cadherin superfamily, presents in fetal liver and gastrointestinal tract during embryogenesis, thus is also named as liver-intestinal cadherin (LI cadherin). CDH17 is overexpressed in hepatocellular carcinoma [5], [6], gastric cancer [7], ductal pancreatic cancer [8] and colorectal cancer [9]–[11]. As reported, CDH17 was mainly present on the cell membrane and absent in normal gastric tissues and the positive rate was nearly 78.4% [12]. The expression level of CDH17 was characteristic of the advanced gastric carcinoma that was associated with poor prognosis [13]; and it was also significantly associated with the lymph node metastasis in gastric cancer [14]. Knockdown CDH17 with lentivirus-mediated miRNA inhibited the proliferation, adherence, tumor growth, and metastasis of BGC823 human gastric cancer cells both in vitro and in vivo [15]–[17]. CDH17 has been proposed as an oncogene and a useful marker for diagnosis of gastric cancers [18]. It has been evidenced that CDH17 mediated oncogenic signaling in HCC is related with Wnt signaling pathway [5]. Recently, it was reported that CDH17 induced tumorigenesis and lymphatic metastasis in GC through activation of NFκB signaling pathway [19]. CDH17 regulated α2β1 integrin signaling to induce specific focal adhesion kinase and Ras activation, which led to the increase in cell adhesion and proliferation in colon cancer cells [11]. However, the main role and signaling mechanism of CDH17 in GC remains unclear. In this study, to validate CDH17 as a potential therapeutic target for GC and to investigate the signaling mechanism of CDH17 in GC, we characterized the expression of CDH17 in human GC cell lines and Chinese GC tissues, checked the influence of CDH17 knockdown or over-expression on tumorigenic and metastatic effect of GC cell lines, and explored the possible signal cascades related to CDH17. We observed a high CDH17 expression in human GC cell lines and Chinese GC tissues, and a clear inhibition in cell proliferation, migration, adhesion, colony formation, apoptosis induction, and cell cycle arrest after silencing of CDH17 in human GC cell lines. Furthermore, our results firstly demonstrate the capacity of CDH17 to regulate the activity of integrin-Ras/Raf/MEK/ERK pathway for cell proliferation in GC, and suggest that CDH17 can serve as an attractive therapeutic target for future research.

## Materials and Methods

### Ethics statement

The use and care of experimental animals was approved by the Institutional Animal Care and Use Committee (IACUC), Roche R&D Center (China). The human GC tissue blocks with corresponding adjacent tissue blocks were obtained from Shanghai Biochip Company, a CRO service company. All human tissues were collected with written consent from source patients. All cell lines were purchased from ATCC, USA, Japanese Collection of Research Bioresources, and Shanghai Institutes of Biochemistry and Cell Biology, Chinese Academy of Science.

### Cell lines and Reagents

All the cell lines from American Typical Cell Collection (ATCC), Japanese Collection of Research Bioresources, and Shanghai Institutes of Biochemistry and Cell Biology, Chinese Academy of Science were maintained in respective growth medium which were recommended by the vendors. PMD-18T-CDH17 plasmid was from Sino Biological Inc. Tetracycline (Tet) was from sigma, Cell Counting Kit-8 was from Dojindo Molecular Tech. Human plasma Fibronectin was from R&D systems. Transwell chamber was from Corning (6.5-mm diameter, 8-μm pore size). CDH17 oligo siRNA was from Genepharma Co. with the sequence 5′AAGGCCAAGAACCGAGUCATT 3′. Scram RNA control (Allstar®) was from QIAGEN.

### Tissue micro array immunohistochemistry

Two hundred and sixteen paraffin embedded gastric cancer tissue blocks together with corresponding adjacent tissue blocks (adjacent tissue was sampled at least 5 cm away from primary tumors) were collected and deposited by Shanghai Biochip Company. Tissues were sectioned and randomly prepared onto 3 micro array slides. The ages of patients were 32–84 years (median age 65) with staging from 1A-4 according to American Joint Committee on Cancer (AJCC) guidelines ver.7. All the cases were diagnosed by two senior pathologists with experienced specialty in gastric cancer pathology. The slides were incubated in blocking serum for 20 min, then covered by 100 μL anti-human Cadherin-17 affinity purified monoclonal Ab, mouse IgG (R&D MAB1032 1∶2000 in TBS/T) at 4°C overnight. The slides were covered by 100 µL DAKO Envision+/HRP, Rabbit/Mouse after being washed thoroughly with TBS. One hundred microliters of substrate-chromogen solution (DAB) were applied to the slides and incubated at room temperature for 10 min. Stained slides were examined microscopically by two immunohistochemistry experts. The normal mouse IgG was used as a negative control. Staining was considered to be negative when no positive cell membrane staining could be identified and positive when more than 10% of tumor cells showed cell membrane-positive immunostaining.

### TR stable cell lines

The pLenti6/TR vector (Invitrogen, Cat. No. V48020) and pLenti3.3/TR vector (Invitrogen, Cat. No. A11114) were used to generate stable cell lines that constitutively express high levels of the Tet repressor. These expression plasmids contain elements that allow packing of the construct into virions and the blasticidin or geneticin resistance marker for selection of stably transduced cell lines. Briefly, 293FT packaging cells (Invitrogen, Cat. No. R700-07) were transfected with the pLenti6/TR or pLenti3/TR and the ViraPower^TM^ Packaging Mix (Invitrogen, Cat. No. K4975-00) to produce lentiviral particles. The lentivirus were used to transduce AGS cells or MGC-803 and the blasticidin (8 µg/ml, Invitrogen, Cat. No. R210-01) or geneticin (200 µg/ml, Invitrogen, Cat. No. 10131-027) was used to select for a stable TR-expressing AGS cells named TR-AGS stable cell line or TR-expressing MGC-803 cells named TR-MGC-803 stable cell line. These TR expressing cell lines were used as hosts for expression constructs that facilitate Tet-regulated expression of CDH17 shRNA (short hairpin RNA) or CDH17 gene, respectively.

### Stable AGS cells with Tet-regulated expression shRNA of CDH17 gene

Four miRNA oligonucleotides targeting CDH17 gene ([Homo sapiens] Gene ID: 1015) were designed using an internet application system (Invitrogen). These double-stranded DNA were ligated into the pcDNA6.2™-GW/EmGFP-miR expression system (Invitrogen, Cat. No. K4936-00) which were then transiently transfected into AGS cells by the Lipofectamine 2000 standard procedures (Invitrogen, Cat. No. 11668-027). According to real-time PCR analysis, the sequence 90-2 (Forward primer:5′-TGCTGTGTACTTCACACCAAACACTAGTTTTGGCCACTGACTGACTAGTGTTTTGTGAAGTACA-3′; Reverse primer: 5′-CCTGTGTACTTCACAAAACACTAGTCAGTCAGTGGCCAAAACTAGTGTTTGGTGTGAAGTACAC-3′) of miRNAs showed the highest efficiency and was chosen to per form BP recombination to obtain pENTR-miRNA. This entry vector was then used in LR recombination with pLenti4/TO/V5 (Invitrogen, Cat. No. K4920-00) to obtain pLenti4/TO/V5-miRNA lentivirus using Gateway® technology (Invitrogen, Cat.No.12535-019). The lentivirus were used to transduce TR-AGS stable cells and 200 µg/ml Zeocin (Invitrogen, Cat. No. R250-05) was used to select for a stable Tet-regulated CDH17 shRNA expression cells named TR-AGS-CDH17_KD stable cell line.

### Stable MGC-803 cells with Tet-regulated overexpression of CDH17 gene

Briefly, CDH17 sequence was amplified from pMD-18T-CDH17 plasmid (Sino Biological Inc.) and subcloned into IRES-EGFP vector to obtain CDH17-IRES-EGFP by using Primers: Forward primer: 5′-GGGGACAAGTTTGTACAAAAAAGCAGGCTTCGCCACCATGATACTTCAGGCCCATCTTCAC-3′; Reverse primer: 5′-TAGGGGGGGGGGAGGGAGAGGGGCGGATCCTCACCTTCTCAGAGGTTTGACTTCA-3′). This construct was used to perform BP recombination to obtain pENTR-CDH17-IRES-EGFP. Then the entry vector and pLenti6.3/TO/V5 (Invitrogen, Cat. No. V370-06) vector were used in LR recombination to obtain pLenti6.3/TO/V5-CDH17-IRES-EGFP. Then the lentivirus were produced to transduce TR-MGC-803 stable cells and 4 µg/ml blasticidin was used to select for a stable Tet-regulated CDH17 gene expression cells named TR-MGC-803-CDH17_Over stable cell line.

### Cell proliferation assay

For siRNA transient transfection assays, cells were seeded in 10 cm dish with a density of 5×10^6^ cells/dish and transfected with 20 nM siCDH17 or scramble siRNA. After 48 h of incubation, the cells were disassociated and transferred to 96-well plate at the density of 3000 cells/well, then incubated for another 72 h. The cell viability was assayed with CCK-8 kit (Donjindo, Japan). For TR-inducible AGS-CDH17_KD stable cells, cells were seeded in 60 mm ×15 mm dish and treated with or without Tet (0, 2.5 and 5.0 µg/ml) for different time. Cells were harvested after 2, 3, 4, 5 and 6 days and the cell number were counted by ScepterTM Handheld Automated Cell Counter (millipore). For proliferation rescue assay, TR-inducible AGS-CDH17_KD stable cells were seeded in 60 mm ×15 mm dish and cultured for 10 days with or without 5.0 µg/ml Tet. For rescue group, the culture medium containing Tet was replaced by fresh medium on day 4, and cells were continued to be cultured to day 10. Cells in each group were harvested on day 2, 4, 6, 8 and 10 for cell number counting and Western blotting analysis.

### Cell migration assay

TR-AGS-CDH17_KD stable cells were seeded in 10 cm dish and treated with or without Tet (5 µg/ml). After 120 h of culture, cells were collected and counted. 0.1 ml of cell suspension (1, 2, 3×10^4^ cells/ml) was added to the upper compartment of the chamber. 0.6 ml 10% FBS medium was added to the lower compartment as a chemo attractant. After 24 h of incubation at 37°C, cells were fixed with 90% EtOH at 4°C for 30 min and then stained with 0.1% crystal violet for 15 min. The non-migrant cells were removed from the upper face of the transwell membrane with a cotton swab. The stained cells were subsequently photographed and counted under light microscopy.

### Cell adhesion assay

TR-AGS-CDH17_KD stable cells were seeded in 10 cm dish and treated with or without Tet (5 µg/ml). Pre-coating of 96-well plates was performed by incubating wells with 25 µg/ml fibronectin at 4°C overnight. To reduce non-specific binding, 1% bovine serum albumin (BSA) was added to each well and incubated for 60 min at 37°C, BSA was washed two times with sterile PBS. 120 h after knockdown CDH17 with Tet, cells were collected and seeded into pre-coated 96-well plates with 4×10^4^ cells/well. 2 h later, non-adherent cells were removed by washing with sterile PBS for twice. The adherent cells were assayed by the CCK-8 kit.

### Colony formation assay

Firstly, 6-well plates were prepared with bottom agar layer (0.6% gel). Then, TR-AGS-CDH17_KD cells or siCDH17 transfected cells (as proliferation assay) were suspended in complete medium containing 0.3%–0.4% Noble agar (Sigma-Aldrich, St. Louis, MO) and seeded into prepared 6-well plate and cultured for 7 or 14 days. Cells were stained with thiazolyl blue tetrazolium bromide (MTT) for 2 h. The number of cell colonies was counted.

### Cell cycle analysis

TR-AGS-CDH17_KD stable cells were seeded in a six well plate at 1.0×10^5^ cells/well and treated with 5 µg/ml Tet. 120 h later, cells were harvested with trypsin and fixed with EtOH at 4°C for 3 h. Then cells were centrifugated at 1,500 g for 5 min and washed with PBS. Re-suspended cells in RNase/PBS solutions (20 µg/ml), incubated at 37°C for 15 min, then added PI/PBS solutions (20 µg/ml) and incubated at RT for 30 min, inspected the stained cells with BD FACS Calibur and analyzed the data with BD CellQuest Pro software.

### Apoptosis analysis

TR-AGS-CDH17_KD stable cells were seeded in a six well plate at 1.0×10^5^ cells/well and treated with 5 µg/ml Tet. 120 h later, cells were harvested with trypsin and washed with pre-cooled PBS once. Cells were re-suspended in 500 µl 1× binding buffer and 5 µL PI and 5 μl AnnexinV (BD) were added. After incubation in dark (room temperature) for 10 min, the stained cells were inspected with BD FACS Calibur. The collected data was analyzed with BD CellQuest Pro software.

### Western blotting analysis

Cells were lysed by RIPA buffer [150 mM NaCl, 1% NP40, 0.25% deoxycholate and 10 mM Hepes (pH 7.4)] containing a protease inhibitor cocktail (Roche Molecular Biochemicals). Protein concentrations were quantified using the bicinchoninic acid (BCA) method (Pierce). Thirty micrograms of protein per sample was boiled in loading buffer and electrophoresed in 8–12% gradient SDS polyacrylamide gels (Invitrogen) and transferred onto nitrocellulose membranes. The membranes were probed with antibodies (CDH17, Integrin β1, Integrin β4, Integrin β5, p-p53, p53, p21 were from R&D, Raf, p-MEK, MEK, p-ERK, ERK were from Cell signaling, and K-Ras was from Santa Cruz) and followed by a peroxidase-conjugated immunoglobulin. Western blots were visualized with an enhanced chemiluminescence (ECL) kit (GE, Healthcare).

### Antibody arrays assay

Three types of antibody arrays were obtained from R&D Systems, Inc. for antibody arrays assay. Human Phospho-Kinase Array Kit (Cat. No. ARY003) contains 43 different kinases. Human Soluble Receptor Array Kit Non-Hematopoietic Panel (Cat. No. ARY012) contains 119 different soluble receptors. Human Apoptosis Array Kit (Cat. No. ARY009) contains 35 different apoptosis antibodies. More exhaustive information about these arrays can be found in the link of R&D Systems: http://www.rndsystems.com/. TR-AGS-CDH17_KD stable cells were harvested after 120 h treated with or without Tet (5 µg/ml) and the proteins were lysed by lysis buffer with the protease inhibitors, cell extracts were diluted and incubated with the three types of antibody arrays overnight. The arrays were washed to remove unbound proteins, followed by incubation with a cocktail of biotinylated detection antibodies. All procedures are according to the instruction of the kit. Blots were analyzed by densitometry, and protein expression was normalized to a positive control which was represented in each membrane.

### Measurement of in vivo activity

TR-MGC803-CDH17_Over cells (1×10^7^ cells in 0.2 ml PBS) were injected into left armpit of 5∼6-week BALB/c nu/nu athymic female mouse (Sino-British SIPPR/BK Lab. Animal Ltd, China). Tumor volume (mm^3^) was measured with calipers and calculated as (W^2^×L)/2, where W is the width and L is the length. When tumor volume reached ∼100 mm^3^, the mice were randomized into two groups (9 mice in each group). Tetracylcine induction (Invitrogen, 0.2 mg/ml, supplied in sterilized drinking water containing 2.5% sucrose) was initialized on the grouping day. Sterilized drinking water containing 2.5% sucrose was supplied to the mice in non-induced group. Tumor volumes were recorded every 3 days until animals were sacrificed. After 35 days induction, xenograft tumor tissues were collected and subjected to Western blot analysis for relative protein expression.

### Statistical analysis

Data were expressed as means ± s.d. and statistically subjected to Student's unpaired t-test. A level of P<0.05 was considered to be significant.

## Results

### Expression profile of CDH17 in gastric cancer

**Figure 1:**
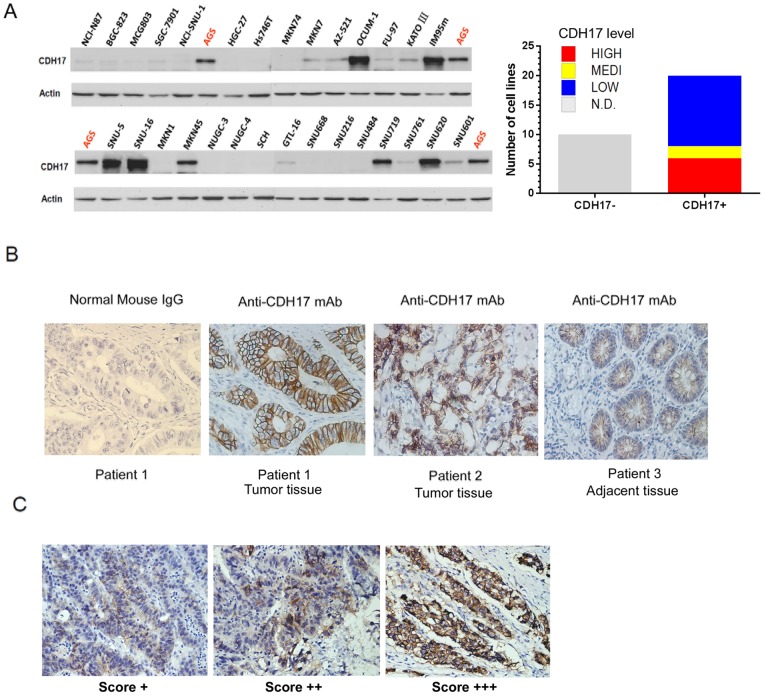
CDH17 expression in a panel of gastric cancer cell lines and in gastric cancer tissue microarray. (**A**) Thirty gastric cell lines are lysed and subjected to Western blot analysis (left). All tested cells are categorized into 4 expression level of CDH17, High, Median (Medi), Low, Not detected (ND), based on optical density analysis against beta-actin (right); (**B**) Representative IHC pictures from patient 1 (well-differentiated gastric cancerous tissue), patient 2 (poorly differentiated gastric cancerous tissue) and patient 3 (adjacent normal tissue); CDH17 showed clear membrane location in tumor or adjacent tissues; (**C**) Representative IHC pictures of positive CDH17 stain with scoring from + to +++.

The expression level of CDH17 proteins in 30 cell lines was categorized into 4 groups based on the data from optical density analysis against beta-actin. AGS cell line was used as internal standard for avoiding the discrepancy between gels. Over 66% of GC cell lines (20/30) were CDH17 positive (Figure 1A).

The expression and localization of CDH17 in primary tissue paraffin section was detected by immunohistochemistry on tissue microarrays. Compared with control slides stained by normal mouse IgG, CDH17 mAb staining was demonstrated clear cell membrane localization (Figure 1B). Among 216 cases with confirmed pathology diagnosis report, 73.6% cancerous tissues (159/216) harbored positive CDH17 stain with scoring from + to +++, while 37% positive CDH17 stain in respective adjacent normal tissues (Table 1). In CDH17 positive cases, the expression level of CDH17 has positive correlation with lymph node metastasis. However, the expression level is reversely associated with gastric wall invasion, tumor distant metastasis, and tumor TNM stages. Our observation was consistent with previous clinical reports [20]–[22]. Among CDH17 positive cases, cancerous tissues exhibited stronger staining of CDH17 (32.1% as scoring +, 25.2% as scoring ++ and 42.8% as scoring +++) than that of adjacent normal tissues (62.5% as scoring +, 21.3% as scoring ++ and 16.3% as scoring +++). While no significant relationship was observed between the stain scoring and clinical stage. Taking all this evidences together, CDH17 can be a diagnostic marker for early stage gastric cancer in Chinese populations.

**Table 1:** Tissue microarray analysis of CDH17 and clinical pathological characteristics in Chinese gastric cancers.

		CDH17 expression				
		Positive				Negative
Factor	No. of Patients	Total: No. of patients (%)	Score+: No. of patients (%)	Score++: No. of patients (%)	Score+++: No. of patients (%)	No. of Patients (%)
**Total**						
Tumor	216	159 (73.6)	51 (32.1)	40 (25.2)	68 (42.8)	57 (26.4)
Perilesional tissue	216	80 (37.0)	50 (62.5)	17(21.3)	13(16.3)	136 (63.0)
**Gender**						
Male	150	114 (76.0)	33 (28.9)	34 (29.8)	47 (41.2)	36 (24.0)
Female	66	45 (68.2)	18 (40.0)	6 (13.3)	21 (46.7)	21 (31.8)
**Tumor size (cm)**						
> = 5.0	128	96 (75.0)	31 (32.3)	24 (25.0)	41 (42.7)	32 (25.0)
<5.0	84	62 (73.8)	23 (37.1)	16 (25.8)	24 (38.7)	22 (26.2)
**Lymph node metastasis**						
N0	53	39 (73.6)	10 (25.6)	10 (25.6)	19 (48.7)	14 (26.4)
N1	38	26 (68.4)	11 (42.3)	5 (19.2)	10 (38.5)	12 (31.6)
N2	58	42 (72.4)	13 (31.0)	15 (35.7)	14 (33.3)	16 (27.6)
N3	60	48 (80.0)	15 (31.3)	9 (18.8)	24 (50.0)	12 (20.0)
**Depth of wall invasion**						
T1	13	12 (92.3)	4 (33.3)	4 (33.3)	4 (33.3)	1 (7.7)
T2	22	17 (77.3)	5 (29.4)	6 (35.3)	6 (35.3)	5 (22.7)
T3	138	100 (72.5)	28 (28.0)	21 (21.0)	51 (51.0)	38 (27.5)
T4	32	22 (68.8)	9 (40.9)	6 (27.3)	7 (31.8)	10 (31.3)
**Distant metastasis**						
M0	201	149 (74.1)	48 (32.2)	38 (25.5)	63 (42.3)	52 (25.9)
M1	15	10 (66.7)	3 (30.0)	2 (20.0)	5 (50.0)	5 (33.3)
**TNM stages**						
I	17	14 (82.4)	3 (21.4)	4 (28.6)	7 (50.0)	3 (17.6)
II	69	50 (72.5)	16 (32.0)	13 (26.0)	21 (42.0)	19 (27.5)
III	100	75 (75.0)	23 (30.7)	17 (22.7)	35 (46.7)	25 (25.0)
IV	15	10 (66.7)	3 (30.0)	2 (20.0)	5 (50.0)	5 (33.3)
**Histological grade**						
I Well differentiated	4	4 (100.0)	1 (25.0)	2 (50.0)	1 (25.0)	0 (0.0)
II Moderatelyl differentiated	93	69 (74.2)	22 (31.9)	16 (23.2)	31 (44.9)	25 (26.9)
III Poorly differentiated	95	68 (71.6)	24 (35.3)	14 (20.6)	30 (44.1)	27 (28.4)

### RNAi-mediated knock down CDH17 on GC cell lines showed suppression of cell proliferation and colony formation in vitro

**Figure 2:**
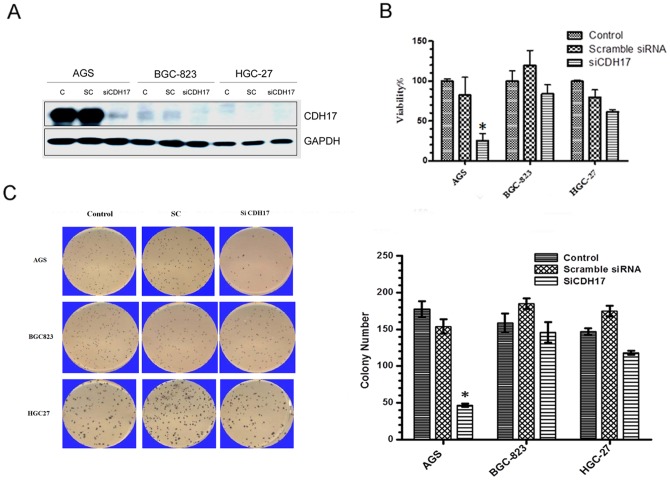
Suppression of *in vitro* cell proliferation and colony formation by CDH17 siRNA. (**A**) The siRNA knock-down efficiency was confirmed by Western blotting 48 h post transfection with 20 nM siCDH17 or scramble siRNA; (**B**) Proliferation assay. The cells were transfected with 20 nM siCDH17 or scramble siRNA in 10 cm dishes. 48 h later, the cells were suspended and reseeded into 96-well plates. The cell viability was assayed with CCK-8 cell proliferation kit 72 h post seeding. The experiment was carried out in triplicate, and the data were presented as mean ± standard deviation; (**C**) Colony formation assay. The cells were transfected with siCDH17 as proliferation assay and reseeded into 6-well plates. The colonies were counted under microscope after stained with MTT 14 days post seeding. The experiment was carried out in triplicate and the typical images were shown. The data were presented as mean ± standard deviation.

Based on the results of CDH17 expression profile in a panel of GC cell lines (Fig 1A), AGS (higher CDH17), BGC-823 (lower CDH17) and HGC-27 (none CDH17) cell lines were selected for further in vitro cellular functional assessment with RNAi-mediated CDH17 silencing. Western blotting showed that AGS cells had high CDH17 protein level and expression of CDH17 was knocked down almost totally by siCDH17. BGC823 cells had low CDH17 protein level and HGC-27 had no CDH17 protein (Figure 2A). Compared with the scramble control cells, CDH17 knocking down had little effect on the proliferative ability of BGC823 and HGC27 cells, whereas had dramatic effect on AGS (by 60% at 72 h, Figure 2B). Soft agar assay showed that the ability to form colony was inhibited in AGS cells if CDH17 was knock down (by 66% at 14 d), but in BGC823 cells and HGC27 cells, CDH17 knocking down had little effect on the colony formation ability (Figure 2C). There was not obvious difference between BGC823 cells (low CDH17 level) and HGC-27cells (non-CDH17) in the inhibition of proliferation and colony formation induced by siRNA CDH17 knockdown.

### Stable cell lines carrying TR inducible knockdown of CDH17 in AGS cells and overexpression of CDH17 in MGC-803 cells

**Figure 3:**
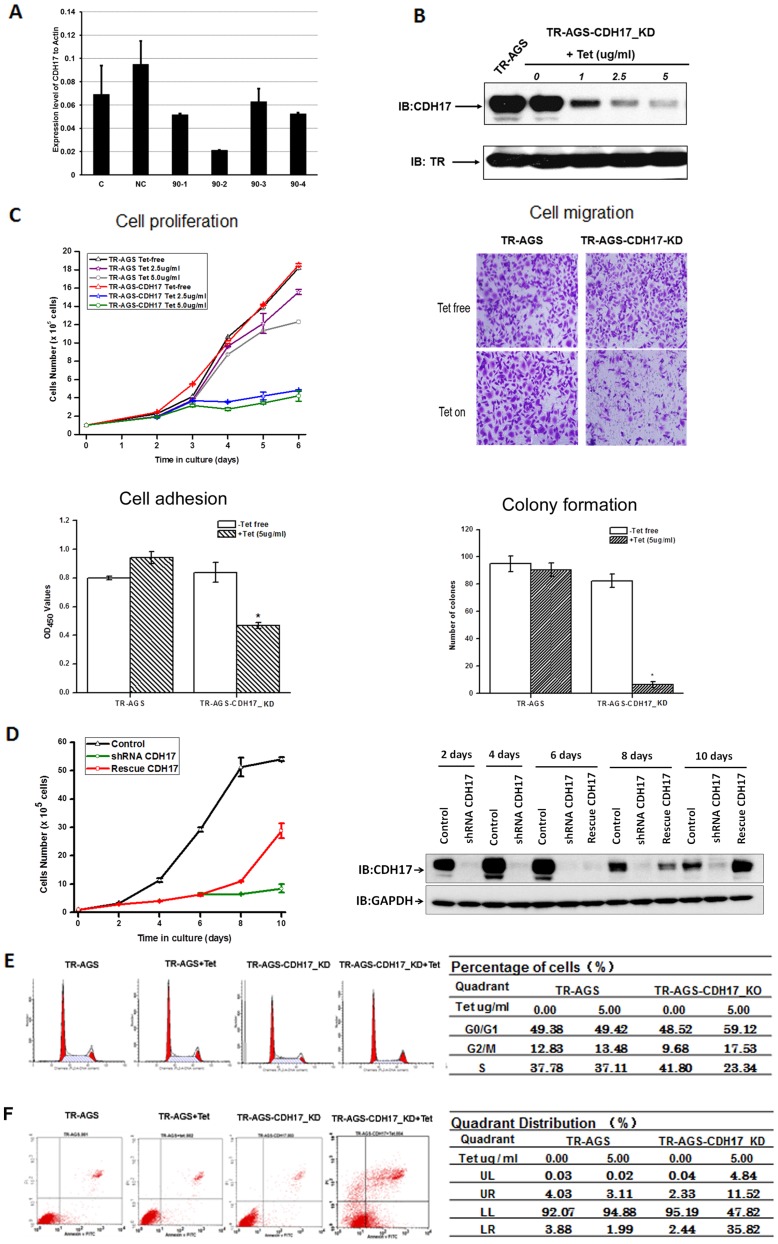
Knockdown of CDH17 in AGS cells inhibited cell proliferation, migration, adhesion, colony formation and induced a cell-cycle arrest and apoptosis. Different assay were described in materials and methods. (**A**) Real-time PCR analysis showed the knock down of CDH17 at the mRNA level in AGS cells (C), AGS cells transiently transfected with pcDNA^TM^6.2-GW/EmGFP-scramble miR plasmid (NC) and pcDNA^TM^6.2-GW/EmGFP-CDH17 miR plasmids (90-1, 90-2, 90-3, and 90-4); (**B**) Western blotting for the effect of CDH17 knockdown using different concentrations of Tet for 48 h in TR-AGS-CDH17_KD cells; (**C**) Cell proliferation, migration (6 days post treatment), adhesion (6 days post treatment. **P<0.01) and colony formation in soft agar (7 days post treatment. **P<0.01); (**D**) Proliferation rescue assay. TR-inducible AGS-CDH17_KD stable cells were seeded in 60 mm ×15 mm dish and cultured for 10 days with or without 5.0 µg/ml Tet. For rescue group, the culture medium containing Tet was replaced by fresh medium on day 4, and cells were continued to be cultured to day 10. Cells in each group were harvested on day 2, 4, 6, 8 and 10 for cell number counting (left) and Western blotting analysis (right); (**E**) Cell cycle analysis after cells were treated with 5.0 µg/ml Tet for 6 days; and (**F**) Cell apoptosis analysis after cells were treated with 5.0 µg/ml Tet for 6 days by flow cytometry.

**Figure 4:**
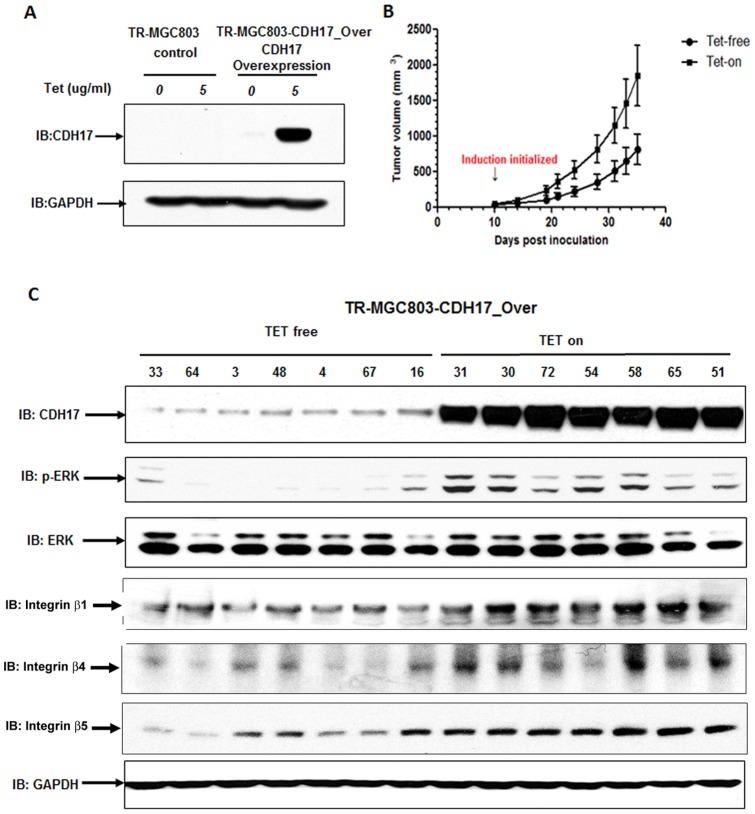
Overexpression of CDH17 in MGC-803 cells promoted tumor growth in nude mice. (**A**) Western blotting showed the induction efficiency of CDH17 overexpression; (**B**) Tumor growth in nude mice. When tumor volume reached ∼100 mm^3^, the drinking water was replaced with 2.5% sucrose containing 0.2 mg/ml Tet or not. *P<0.05, Tet-on group vs Tet-free group; (**C**) Western blotting analysis of xenograft tumor tissues.

The target sequence in exon13 (90-2) yielded above 90% reduction in mRNA level (Figure 3A). Then we used pLenti4/TO/V5-miRNA-90-2 CDH17 lentivirus to transduce TR-AGS stable cells and got a stable Tet-regulated expression shRNA of CDH17 gene cells (TR-AGS-CDH17_KD) which the CDH17 protein level was reduced about 90% after induced with 5 µg/ml Tet (Figure 3B). The pLenti6.3/TO/V5-CDH17-IRES-EGFP lentivirus was used to transduce TR-MGC-803 stable cells and selected with blasticidin to get a stable Tet-regulated expression CDH17 gene cells (TR-MGC-803-CDH17_Over). The overexpression of CDH17 in this stable cell was confirmed at the protein level by Western blotting (Figure 4A).

### Knockdown of CDH17 induced inhibition in cell proliferation, migration, adhesion, colony formation and induction in cell-cycle arrest and apoptosis

The TR-AGS cells (control cells) and TR-AGS-CDH17_KD cells were used to assess the potential cellular functional effects of shRNA-mediated CDH17 knockdown in AGS cells. TR-AGS-CDH17_KD cells treated with 5 µg/ml Tet showed a reduction in cell proliferation (by 75.8% at 5 days), cell migration ability (by 68.1% at 16 h), cell adhesion ability (by 46.8% at 30 minutes), and colony forming ability (by 92.7% at 7 days) compared with Tet free (Figure 3C). In proliferation rescue study, CDH17 knockdown in TR-AGS-CDH17_KD cells induced by Tet can be rescued by removing Tet. After removing Tet on day 4, re-expression of CDH17 was observed on day 8 and 10 of culture in CDH17 shRNA knockdown AGS cells. Re-expression of CDH17 could rescue the proliferation ability of TR-AGS-CDH17_KD cells (Figure 3D). Compared to Tet free TR-AGS-CDH17_KD cells, Tet on cells demonstrated a significantly increased percentage of cells in G0/G1 and G2/M phase and a dramatically deceased percentage of cells in S phase (Figure 3E). In the meantime, we also observed that the number of apoptotic cells (by 52.2% at 5 days) was increased by down-regulation of CDH17 in TR-AGS-CDH17_KD cells (Figure 3F). No significant difference was found in TR-AGS stable cell line with or without Tet incubation.

### Overexpression of CDH17 promoted the growth of tumor xenografts in nude mice

TR-MGC-803-CDH17_Over cells were injected subcutaneously into the flanks of BALB/c nude mice to form solid tumor. The expression of CDH17 was under the control of Tet in drinking water. Results showed that after 35 days induction, Tet-induced group (tumor volume  = 1849.08±423.46m3) exhibited a 2.27-fold higher growth progress rate versus Tet-free group (tumor volume  = 814.04±234.69m3) (p<0.05) (Figure 4B), and induced CDH17 expression in tumor tissues can be analyzed by Western blotting (Figure 4C). It indicated that CDH17 plays an important role in the carcinogenesis of gastric cancer. We also used the TR-AGS-CDH17_KD cells to observe whether silencing of CDK17 may affect the tumorigenicity in nude mice. Unfortunately, the tumor take rate of this cell line was very low and no reliable in vivo results were observed with TR-AGS-CDH17_KD cell line.

### Determination the related protein levels after knockdown CDH17 by Antibody arrays assay

**Figure 5:**
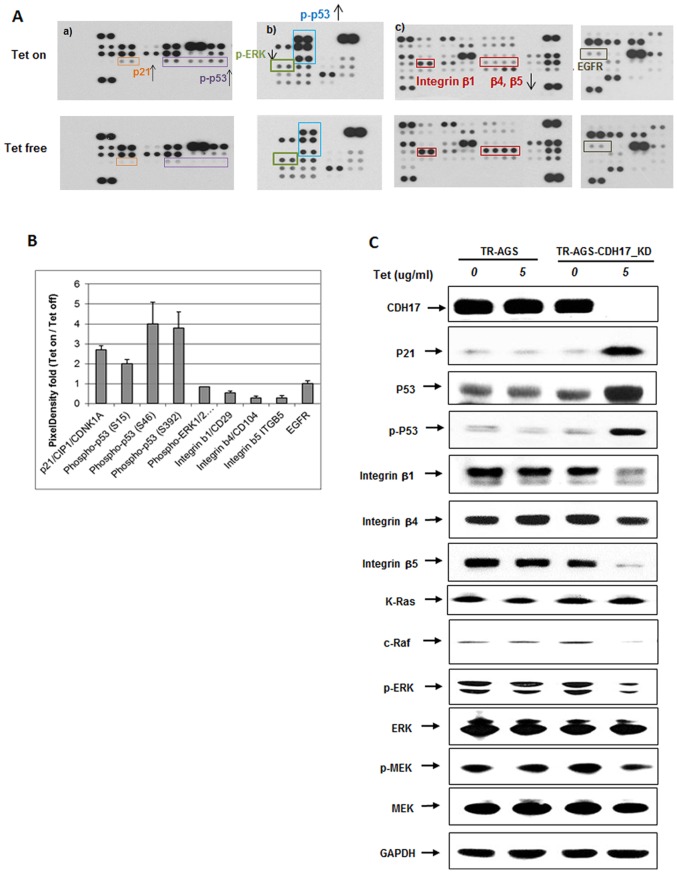
Knockdown CDH17 in AGS cells induced alteration of related proteins. TR-AGS-CDH17_KD stable cells were treated or untreated with 5 µg/ml Tet for 5 days. (**A**) Antibody array assay. (a) Human Apoptosis Array Kit, (b) Human Phospho-Kinase Array Kit, (c) Human Soluble Receptor Array Kit Non-Hematopoietic Pane; (**B**) Changing ratio of related proteins between Tet-on and Tet-free cells; (**C**) Changes of related proteins found in antibody array assay were confirmed using Western blotting analysis.

According to the tumorigenic effects (inhibition of cell proliferation, colony formation, and tumor growth in vivo) and metastatic effects (inhibition of cell migration and adhesion) of CDH17 knockdown, we choose three antibody arrays (from R&D systems) to test the relative protein changes and hope to find potent CDH17 regulated related proteins. In the apoptosis array, phospho-p53 (S15, S46 and S392) and p21/CIP1/CDNK1A expression increased about two fold after 5 µg/ml Tet treatment for 5 days compared to untreated cells (Figure 5A (a) and 5B). In the phospho-kinase array, phospho-p53 (S15, S46 and S392) expression also increased after Tet treatment and, at the same time, expression of phospho-ERK1/2 (T202/Y204) decreased (Figure 5A (b) and 5B). In the soluble receptor array, knockdown of CDH17 in AGS cells could significantly decrease the expression of integrin β1, β4, β5, while did not impact the expression of EGFR (Figure 5A(c) and 5B). The alteration ratios of interested proteins between Tet-on and Tet-free AGS cells were summarized in Figure 5B.

### Knockdown of CDH17 in AGS downregulated β-integrins and Ras/Raf/MEK/ERK signaling pathway

The protein changes from antibody array assay were confirmed by Western blotting analysis. The results indicated a significant reduction of integrin β1, β4, β5 and phospho-ERK in CDH17 knockdown AGS cells, but no significant change in the total ERK (Figure 5C). Then we evaluated the involvement of other Ras/Raf/MEK/ERK signaling pathway components. Knockdown of CDH17 resulted in down-regulation of the Raf, phospho-MEK, and phospho-ERK proteins (Figure 5C). From TR-MGC-803-CDH17_Over in vivo study, we observed the overexpression of CDH17 enhanced the integrin β1, β4, β5 expression and ERK activation (Figure 4C) and it is consistent with above findings. To investigate whether the effect of CDH17 on integrins or Ras protein is direct interaction or not, a co-immunoprecipitation of CDH17 with integrin β1, integrin β4, integrin β5, integrin α2, and Ras was conducted in gastric cancer AGS cells, respectively. Out of expectation, in contrast to Bartolome's result [11], there was no direct association between CDH17 and any one of these integrins or Ras protein (data not shown).

## Discussion

It is a great challenge to clinicians and basic scientists to determine the molecular markers associated with the progression and prognosis of related cancers. In liver and gastric cancers, it is believed that the high expression of CDH17 was associated with poor survival, tumor recurrence, tumor invasion, metastasis and advanced tumor stage [23]–[25]. In current study, among 30 panels GC cell lines, over 66% of our tested cell lines (20/30) are CDH17 positive, and nearly half of these cell lines have higher CDH17 expression. In our pathology diagnosis report, 73.6% Chinese GC cancerous tissues (159/216) harbored positive CDH17 stain. In CDH17 positive cases, the expression level of CDH17 has positive correlation with lymph node metastasis. However, the expression level is reversely associated gastric wall invasion, tumor distant metastasis, and tumor TNM stages. Our observation was consistent with previous clinical reports [20]–[22]. Taking all this evidences together, CDH17 can be a diagnostic marker for early stage gastric cancer in Chinese populations.

To identify the effect of CDH17 in gastric cancer tumorigensis and metastatic potential, both oligo siRNA and TR inducible pLentiviral vectors with specific miRNA (exton13) against CDH17 were used for knock-down CDH17 expression in gastric cancer cell lines. CDH17 silencing by oligo siRNA could inhibit cell proliferation and colony formation in GC cell lines with high CDH17 expression. The inhibitory effect was dramatic on AGS which has a higher CDH17 expression, but not on BGC823 and HGC27 cells which have very low or no expression of CDH17 protein (Figure 2). It indicated that the CDH17 expression may contribute to cancer cell proliferation and colony formation ability in AGS cells. Furthermore, similar results were also observed in tet-induced shRNA-mediated CDH17 knockdown in AGS cells which resulted in inhibition of cell proliferation, migration, adhesion, colony formation and induction of apoptosis and cell cycle arrest in vitro. Re-expression of CDH17 could rescue the proliferation ability of TR-AGS-CDH17_KD cells (Figure 3). In addition, Tet induced over-expression of CDH17 in TR-MGC803-CDH17_Over cells could promote the growth of tumor xenografts in vivo (Figure 4). Taking all this evidences together, we can conclude that there is a significant correlation between CDH17 expression and the tumorigenic and metastatic abilities of gastric carcinoma, and CDH17 can serve as a potential therapeutic target for future research.

Although several previous studies had reported the fact that silencing of CDH17 could inhibit the cell proliferation in GC cells, the signaling mechanism of CDH17 remains unclear. It has been evidenced that CDH17 mediated oncogenic signaling in HCC is related with Wnt signaling pathway [5]. However, there were some differences between our results and the results reported by Liu in HCC. Our results showed no change in phosphorylated form of glycogen synthase kinase 3β (GSK-3β and the retaining of β-catenin in the nucleus after silencing CDH17 (data not shown). This discrepancy could be explained by the utilization of different cancer cell type and indicates significant differences in the molecular mechanism of action of CDH17 between GC and HCC. Recently, Wang J et al. investigated the relationship between CDH17 and lymph node micrometastasis (LNMM). Their study showed that CDH17 induced tumorigenesis and lymphatic metastasis in GC through activation of NFκB signaling pathway [18]. To further explore alternative pathways other than β-catenin/Wnt and NFκB signaling pathway to explain the observed phenotypes, we used a “high throughput” antibody array assay to test the protein level change between Tet free and Tet on TR-AGS-CDH17_KD stable cells. After confirming the altered expression level of proteins by Western blotting analysis, we found that the changes of relevant proteins level in CDH17 knockdown AGS cells as below: phosphate-ERK (↓), phosphor-p53 (↑), p53 (↑), p21 (↑), and integrin β series proteins (↓) (Figure 5). It was interesting for us to further evaluate the involvement of other Ras/Raf/MEK/ERK signaling pathway components. Further data showed that knockdown of CDH17 resulted in down-regulation of the Raf, phospho-MEK, and phospho-ERK (Figure 5C). Consistent with the above findings, up-regulation of the phospho-ERK and integrin β series proteins was observed in MGC-803 cells with over-expression of CDH17 (Figure 4C).

The Ras/Raf/MEK/ERK mitogen-activated protein kinase (MAPK) cascade is a key intracellular signaling pathway that regulates diverse cellular functions including proliferation, survival, apoptosis, motility, transcription, metabolism and differentiation [26], [27]. The MAPK pathway has a well-defined role in cancer biology and has been an important target in the development of targeted therapies [28]. In our study, knockdown of CDH17 was able to inactivate the Ras/Raf/MEK/ERK signaling pathway in AGS cells and lead to less tumorigenic and metastatic activities of cells in vitro. While, over-expression of CDH17 was able to activate Ras/Raf/MEK/ERK signaling pathway in MGC-803 cells and promote the tumorigenicity of cells in vivo.

In addition, knockdown of CDH17 enhanced both p53 and phospho-p53 activation, and combined with the increasing of p21 protein level (Figure 5). P53 dependent accumulation of p21 has been shown to mediate both G1 phase arrest and G2-M phase progression and induce cell apoptosis [29], [30]. In this study, the cell cycle arrest and apoptosis induced by CDH17 knockdown (Fig 3E, 3F) might be attributed to its up-regulation of phosphor-p53, total p53 and p21 expression. It has become clear that the MAPK pathway can functionally interact with p53 protein [31]. The Ras/Raf/MEK/ERK pathway has different effects on growth, prevention of apoptosis, cell cycle arrest and induction of drug resistance in cells of various lineages which may be due to the presence of functional p53 [27]. It was suggested that the up-regulation of p53 and p21 in CDH17 silencing AGS cells might be resulted from the modulation of Ras/Raf/MEK/ERK pathway.

The Ras/Raf/MEK/ERK pathway is activated by diverse mechanisms. MAP kinases are activated in response to many different signals including those originating at growth factor receptors such as epidermal growth factor (EGF) and cell adhesion receptors such as the integrins [26], [32]. In our results, we found that knockdown of CDH17 in AGS cells could significantly decrease the expression of integrin β1, β4, β5, while did not impact the expression of EGFR (Figure 5A(c) and 5B). The results suggested that the knockdown of CDH17 inactivated Ras/Raf/MEK/ERK pathway might be through its reduction of integrins on cell membrane, not through the EGF/EGFR signaling. Since both CDH17 and integrin are cell surface adhesion molecules, there might be direct interaction between CDH17 and integrins. Bartolomé et al demonstrated an interaction between CDH17 and α2β1 integrin with a direct effect on β1 integrin activation in colorectal cancer cells. CDH17 modulated integrin activation and signaling to induce specific focal adhesion kinase and Ras activation, which led to the increase in colorectal cancer cell adhesion and proliferation [11]. In current study, a co-immunoprecipitation of CDH17 with integrin β1, integrin β4, integrin β5, and integrin α2 was conducted in gastric cancer AGS cells, respectively. Out of expectation, there was no direct association between CDH17 and any one of these integrins (data not shown). The reason to explain this phenomenon is that the interaction between CDH17 and α2β1 integrin is cell type dependent, which specifically exists in colorectal cancer cells, but not in gastric cells. The direct interaction between CDH17 with other proteins still needs to be further investigated in the future.

**Figure 6:**
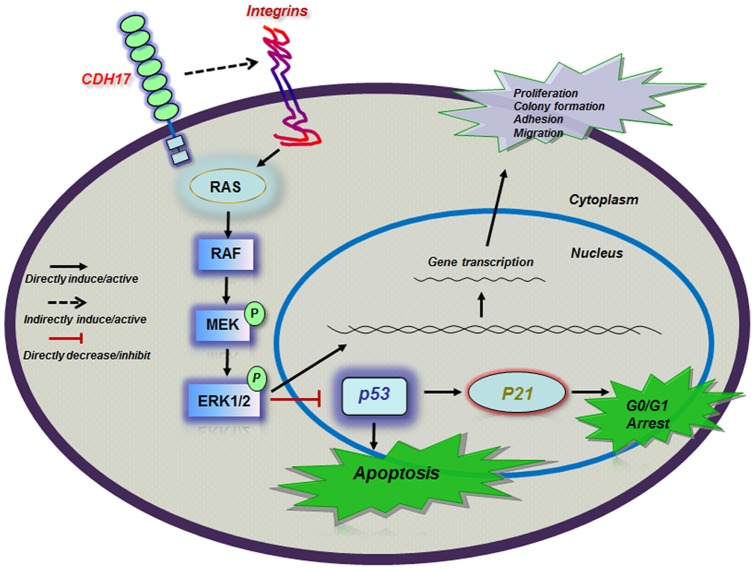
Schematic diagram of the regulatory and signaling network of CDH17 in GC. This schematic diagram demonstrates the inducing effect of CDH17 on Ras/Raf/MEK/ERK signaling pathway and illustrates the hypothetic involvement of integrins in GC. CDH17 indirectly affects integrins to stabilize their structure and activity. The up-regulation of cadherin-integrin signaling activates the Ras/Raf/MEK/ERK pathway. The activation of ERK regulates various nuclear and cytoplasmic substrates, including p53 and p21, which involve in diverse cellular responses, such as cell proliferation, migration, adhesion, colony formation, cell-cycle and apoptosis.

Together, our current observations point to a potential oncogenic role for CDH17 in GC and also propose a novel mechanism of action of CDH17. In the proposed model (Figure 6), we hypothesize that CDH17 may (1) indirectly affect integrins to stabilize their structure and activity, (2) activate the Ras/Raf/MEK/ERK pathway by up-regulation of cadherin-integrin signaling, (3) retain p53 and p21 at a lower level through activation of Ras/Raf/MEK/ERK pathway (4) play an important role in cell proliferation, migration, adhesion, colony formation, cell-cycle and apoptosis.

In conclusion, the present study identifies that CDH17 is an actual oncogene and plays a major role in cell proliferation and tumor growth in GC through integrin-Ras/Raf/MEK/ERK signaling. It also presents a novel therapeutic approach against GC by targeting CDH17.
